# Raspberry protective role in inflammatory diseases: An overview

**DOI:** 10.22038/ijbms.2025.89042.19218

**Published:** 2026

**Authors:** Priyanka Arya, Vikram Sharma, Priyanka Singh, Rahul Sagar, Surabhi Thapliyal, Manu Sharma

**Affiliations:** 1 Galgotias College of Pharmacy, Greater Noida, U.P., India; 2 Banasthali Vidyapith, Department of Pharmacy, Rajasthan, India; 3 AnovIP, New Delhi,110049, India; 4 Department of Pharmacology, All India Institute of Medical Sciences, Rishikesh 249203, India

**Keywords:** Alzheimer’s, Atherosclerosis, Cancer, Cardiovascular, Inflammation, Neuroinflammation, Parkinson’s, Raspberry

## Abstract

Inflammation is a natural immune response triggered by multiple factors such as pathogens, damaged cells, and toxic substances. These triggers can lead to both acute and chronic inflammatory reactions in different tissues, contributing to the development of several inflammatory disorders, including cardiovascular diseases, neuroinflammation, arthritis, and cancer. Both infectious and non-infectious stimuli activate immune cells and initiate critical inflammatory signaling pathways.

Raspberries (*Rubus idaeus*) are abundant in bioactive constituents, especially polyphenols like anthocyanins, flavanols, phenolic acids, urolithin A, and ellagic acid, all of which possess notable anti-inflammatory and anti-oxidant activities. These compounds have been shown to regulate various inflammatory signaling pathways, including MAPKs, NF-κB, PI3K/Akt, AP-1, IL-6, TNF-α, IL-1β, CD40, nitric oxide (NO), caspases, and the JAK-STAT pathway. Studies have emphasized their broad pharmacological effects, such as anti-inflammatory, anti-oxidant, hepatoprotective, cardioprotective, gastroprotective, anti-obesity, skin depigmenting, and bone-regenerative properties. This review emphasizes mechanistic insights into raspberries’ protective roles in managing inflammatory-related disorders, particularly cardiovascular diseases, neurodegenerative conditions, and cancer, and highlights their therapeutic potential.

## Introduction

The immune system regulates the inflammatory response and involves a series of signaling steps. When tissue damage or pathogen infection triggers an immune response, the immune system works to balance an excessive proinflammatory response (1). In reaction to antigens or tissue injury, inflammation is one of the immune system’s initial responses, involving cells from both the innate and adaptive immune systems. At the cellular level, early on, after recognizing a danger signal, a series of molecular events within inflammatory cells leads to the production of inflammatory mediators (2).

When tissue damage or pathogen infection occurs, a signaling cascade is triggered, leading to inflammation. This process begins when pathogen-associated molecular patterns (PAMPs) and damage-associated molecular patterns (DAMPs) recognize and bind to Toll-like receptors (TLRs), which are located in the cytoplasm, endosomes, and plasma membrane, as well as to telencephalin (TLN) cells that are part of the immune system. Once this recognition occurs, the source of the stimulus determines the duration and nature of the inflammatory response, which is usually temporary (3). However, in chronic diseases, the inflammasome can remain activated for extended periods, contributing to cardiovascular disease, neuroinflammation, diabetes, and cancer development. The inflammasome is a cytosolic multiprotein complex that triggers multiple pathways, including MAPKs, NF-κB, PI3K/Akt, AP-1, JAK-STAT, and caspases. It reflects an inflammatory response by activating proinflammatory agents IL6, TNF-α, IL-1β, CD40, and NO (4).

Raspberries are part of the rose family (Rosaceae) and belong to the genus Rubus, which also includes other bramble fruits like blackberries, dewberries, and boysenberries. The scientific name for red raspberries is Rubus idaeus (5). Raspberries are an excellent natural source of (poly)phenols with anti-inflammatory properties, including ellagic acid, flavanols, and phenolic acids. They are also rich in beneficial compounds, such as β-sitosterol, vitamins C, E, and folate (6). Researchers revealed pleiotropic pharmacological effects, anti-inflammatory, anti-oxidant, hepatoprotective, cardioprotective, neuroprotective, antiproliferative, gastroprotective, anti-obesity, depigmentation, bone regeneration, etc (7). A human clinical trial has demonstrated that consuming raspberries can enhance vascular endothelial function, lower total cholesterol, and reduce inflammatory cytokine levels in individuals with metabolic syndrome (8). Researchers reported that when the polyphenolic-enriched red raspberry extract was given as a treatment to the adjuvant-induced arthritis rats, it was revealed that there was a significant reduction in inflammation, decreasing IL-1β level, and improving cartilage damage (67%) and bone resorption. Researchers also report that digested raspberries contain polyphenols, which are considered a good constituent for brain health, mainly by attenuating neuroinflammation through their anti-inflammatory properties (9). Raspberries play a potential role in combating neuroinflammation due to their rich composition of bioactive compounds like polyphenols, particularly ellagic acid and ellagitannins. These compounds possess potent anti-oxidant and anti-inflammatory properties (10). Research suggests that the polyphenols in raspberries can help reduce inflammation in the brain by inhibiting the activation of microglia, the immune cells responsible for neuroinflammatory responses (11- 13). Raspberry polyphenols have demonstrated the ability to inhibit critical inflammatory signaling pathways, including MAPK and NF-κB, while simultaneously enhancing the production of anti-inflammatory cytokines such as IL-10. This combined effect, reducing proinflammatory mediators and increasing anti-inflammatory and anticancer responses, underscores the potential of raspberries in lowering the risk of neurodegenerative disorders associated with chronic neuroinflammation, such as Alzheimer’s and Parkinson’s diseases, as well as cancer (14). This review aims to explore the potential protective mechanisms of raspberry in inflammatory diseases. The utility of this research lies in highlighting the therapeutic potential of raspberry as a natural anti-inflammatory agent, which could be valuable in developing dietary or pharmacological strategies for managing inflammatory diseases such as cardiovascular, neurodegenerative, and cancer.

### Methodology

Scientific literature was gathered using online search engines and databases such as Scopus, PubMed, ScienceDirect, and Google Scholar. The search included articles published between January 2000 and December 2024. Articles were selected based on their relevance to the protective effects of raspberries in inflammatory diseases. The inclusion criteria comprised peer-reviewed original research articles, systematic reviews, and both clinical and experimental studies that investigated the role of raspberry in modulating inflammatory pathways, including NOX1, ROS, P-AKT/P-P38/ERK1/2, NF-κB, NLRP3, p38 MAPK, and ERK. Eligible studies included those conducted on human subjects, in animal models, or *in vitro *systems related to conditions such as neurodegenerative disorders, cardiovascular diseases, and cancer. Search keywords included terms such as “Raspberry,” “Neuroinflammation,” “cardiovascular diseases,” “Cancer,” “Angiotensin,” “Alzheimer’s,” “Atherosclerosis,” “Astrocyte,” “Glial,” “Myocardial Infarction,” “Parkinson’s,” “Breast cancer, “and “Prostate Cancer”. Exclusion Criteria included non-peer-reviewed articles, studies not related to inflammation, keywords unrelated to the topic, or publications in languages other than English.

### Raspberry overview

Raspberries (*Rubus idaeus* L.), part of the rose family, genus *Rubus*, are available in a variety of colors, including red, black, yellow, pink, and purple. The genus *Rubus* encompasses 12 subgenera, over 500 species, and thousands of cultivars, with red raspberries being the most common cultivated variety, followed by black raspberries (15). Red raspberries are cultivated more extensively than black raspberries due to their superior disease resistance and higher productivity compared to black raspberry plants. Berry fruits, particularly raspberries, are regarded as delightful, revitalizing, and flavorful, providing energy and supporting a healthy diet. The plant’s various parts have been used for medicinal purposes as well as for food. For many ages, the fruit, leaves, and blooms of the raspberry have been utilized medicinally (16). Traditionally, raspberry leaves have been used to cure a variety of illnesses, including diabetes, fever, flu, respiratory conditions, gastrointestinal issues, heart issues, morning sickness, and childbirth. Raspberry leaves have been used to stimulate bile production, perspiration, and urination, while red raspberry leaves are applied topically to treat skin rashes and sore throats. Raspberry blossoms have also been used in eye ointments and for treating stomach issues (17).

### Nutritional profile of raspberries

Numerous health-promoting nutrients, such as dietary fibers, fatty acids, and vital vitamins and minerals, are found in red raspberries. Raspberries only contain 52 calories per 100 grams, making them a low-calorie fruit. Their high dietary fiber content (6.5 g/100 g) and significant fructose levels (over 50% of total sugars) help regulate blood sugar by slowing digestion. This, together with their naturally sweet taste, makes them an ideal and healthy alternative to processed meals. Since raspberry seed oil is 97.8% unsaturated and has a low n-6/n-3 fatty acid ratio of 1.64, it is an excellent source of vital, healthful lipids (18). Raspberry seeds contain fat-soluble vitamins such as tocopherols and carotenoids, while the fruit’s flesh is rich in water-soluble vitamin C, providing 26.2 mg per 100 grams of fresh fruit. Raspberry seeds contain fat-soluble vitamins such as tocopherols and carotenoids, while the fruit’s flesh is rich in water-soluble vitamin C, providing 26.2 mg per 100 grams of fresh fruit. They also contain some essential nutrients, including calcium, zinc, thiamine, vitamin B6, riboflavin, and vitamin A (19).

### Bioactive compounds of raspberry

A study by Chen *et al*. (2013) evaluated the phytochemical properties of 15 commercially available raspberry varieties and compared them to their anti-oxidant capacity (20). A more potent anti-oxidant activity was found to correlate with higher phytochemical concentrations. The main phytochemicals found in raspberry are polyphenols, which include phenolic acids, anthocyanins (flavonoids), catechins (flavonoids), proanthocyanidins (flavonoids), and ellagitannins (tannins) (21).

### Ellagitannins and ellagic acid

Raspberries are rich in ellagitannins, a type of tannin that the body converts into ellagic acid upon consumption. Ellagic acid is recognized for its significant health benefits. Sanguiin H-6 and Lambertianin C are the two primary ellagitannins found in raspberries. The most prevalent, Sanguiin H-6, has strong anti-oxidant capacity that may help prevent cancer, while Lambertianin C also possesses potent anti-oxidant effects (22, 23). Because ellagic acid is a potent anti-oxidant, it helps the body fight off free radicals and minimize oxidative stress. Ellagic acid has also demonstrated anticancer properties, shown to inhibit the growth of cancer cells and even induce their death in certain cancers, such as breast, colon, and prostate cancer. Furthermore, Ellagic acid exhibits anti-inflammatory effects, which help prevent long-term diseases such as cardiovascular disorders. As ellagitannins are broken down in the digestive tract, gut bacteria further break down ellagic acid into urolithins, especially urolithin A. These substances offer unique health benefits, such as anti-aging and anti-inflammatory properties (24).

### Anthocyanins

The pigments that give raspberries their deep red, purple, and occasionally blue hues are called anthocyanins, and they constitute a significant class of polyphenols found in the fruit. The two most prevalent anthocyanins found in raspberries are Cyanidin-3-O-glucoside, which contributes to the fruit’s vivid red color and anti-oxidant qualities, and Cyanidin-3-O-sophoroside, which is the most prevalent and has potent anti-oxidant qualities. The presence of pelargonidin derivatives in certain raspberry types can also slightly alter the fruit’s colour (25).

From a health perspective, anthocyanins are potent anti-oxidants that neutralize free radicals and alleviate oxidative stress. Anthocyanins have anti-inflammatory properties that reduce inflammation in the body. They promote heart health by improving blood vessel function, reducing the risk of atherosclerosis, and protecting neurons, potentially lowering the risk of neurodegenerative diseases (26, 27).

### Proanthocyanidins (Condensed tannins)

Shi *et al*. recently isolated proanthocyanidins from raspberries and assessed their anti-oxidant capacity. Condensed tannins, another name for proanthocyanidins, are oligomers or polymers of flavan-3-ols. They are a significant family of polyphenols that give raspberries their anti-oxidant and astringent qualities. The powerful anti-oxidant properties of epicatechins and catechins, which make up proanthocyanidins, are well-known. They offer potential anti-aging benefits and help protect cells from oxidative damage (28).

### Flavonols

Raspberries contain flavonols, a subclass of flavonoids with anti-inflammatory and anti-oxidant properties. They are essential for the fruit’s defense against environmental stressors and also contribute to the health benefits linked to raspberry consumption. The main flavonoids present are quercetin, kaempferol, and myricetin (29). Quercetin improves blood vessel function, which may enhance cardiovascular health by lowering oxidative stress. Kaempferol is known to have anti-inflammatory, anticancer, and anti-oxidant attributes, whilst myricetin has both anti-inflammatory and neuroprotective effects in addition to anti-oxidant benefits (30).

### Raspberry’s potential role in cardiovascular diseases

**Figure1:**
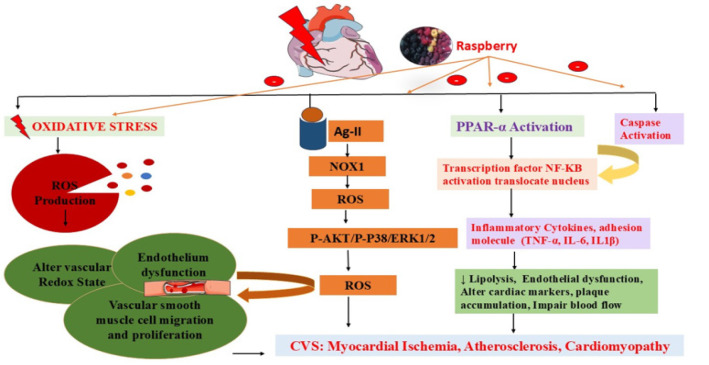
Mechanistic insight into the role of raspberry in cardiovascular diseases

The burden of cardiovascular diseases is increasing rapidly every year. Despite lifestyle modification and advanced therapeutic approaches, the figure for mortality globally per year is 17.5 million (31). The inflammation triggers the oxidative stress processes, which play a critical role in the arterial wall or systemic circulation, primarily triggered by the immune system and modified lipoproteins, resulting in atherosclerotic lesions, myocardial infarction, stroke, etc., cardiovascular diseases. These processes often lead to a hypercoagulable state, contributing significantly to the disease’s clinical complications (Figure 1) (32, 33).

Oxidative stress is defined by a disruption in the balance between reactive oxygen species (ROS) and the body’s anti-oxidant defense (34, 35). This imbalance heightens the likelihood of oxidative damage to cellular components, including DNA, proteins, and lipids, which can impair cellular function, induce mutations, or cause cell death. Elevated oxidative stress, leading to oxidative damage, has been linked to the onset, progression, and complications of cardiovascular diseases (36, 37). Various oxidants within the vessel wall can arise from both cellular and extracellular sources, as well as from enzymatic and non-enzymatic pathways (38).

The vascular endothelium plays a crucial role in maintaining vascular homeostasis, with responsibilities including regulating thrombosis, fibrinolysis, angiogenesis, and vascular tone (39). Nitric oxide (NO), produced from L-arginine through endothelial nitric oxide synthase (eNOS), is a key mediator of these processes. A significant factor contributing to endothelial dysfunction is the reduced availability of NO (40, 41). One widely accepted mechanism for this dysfunction involves excessive reactive oxygen species (ROS), which degrade NO and disrupt eNOS/NO signaling. Reduced NO availability can lead to vasoconstriction and ischemia and promote vascular inflammation, leukocyte adhesion, and the formation of foam cells, which are precursors to atherosclerotic plaques (42). Thus, strategies aimed at reducing ROS production or protecting NO from oxidative damage are essential for preserving endothelial balance and function (Figure 1).

Khan and coworkers in 2018 reported that when rats were treated with isoproterenol, inflammation and oxidative reaction accelerate, which leads to myocardial infarction. In rats’ treatment with raspberry (100 and 200mg/kg) results in cardioprotective effect by reducing cardiac marker level creatine kinase-MB, lactate dehydrogenase, total cholesterol, triglycerides, decreasing low-density-lipoprotein, very-density-lipoprotein, and increasing high-density-lipoprotein. In addition, it also affects the anti-oxidant markers, decreasing malondialdehyde and increasing glutathione, superoxide dismutase, catalase, Na+, and K+ -ATPase. Moreover, it also decreases the nitric oxide, tumor necrosis factor-α, and Nitric oxide synthase (iNOS) level. Histopathological analysis of cardiac tissue reveals that raspberry treatment reduces necrosis, edema, and infiltration of inflammatory cells with improvement in myofibrillar structure to near normal levels (Table 1). They concluded that raspberry treatment found cardioprotective action by suppressing oxidative and inflammatory pathways in myocardial infarction (Figure) (43).

Yu *et al*. reported that *in vitro *studies and animal models have explored the effects of red raspberry polyphenols or red raspberry fruit/extracts on factors relevant to endothelial function. Among these, ellagic acid has been the primary focus in *in vivo* research. Researchers revealed that ellagic acid, at concentrations between 0 and 50 mM, effectively reduced the production of reactive oxygen species (ROS) in human umbilical vein endothelial cells in a dose-dependent manner. Moreover, ellagic acid blocked the IL-1β-induced nuclear translocation of NF-κB, thereby decreasing the expression of vascular cell adhesion molecule-1 (VCAM-1) and E-selectin. This led to a reduction in monocyte adhesion (Table 1).

 These findings indicate that ellagic acid possesses anti-inflammatory properties and may contribute significantly to the prevention of atherosclerosis (44).

Cardiac hypertrophy refers to the enlargement or thickening of the heart muscle, often due to increased workload or stress. This condition can occur due to factors like hypertension, valve disease, or other conditions that force the heart to work harder (45). While initially considered a compensatory mechanism to improve heart function, prolonged hypertrophy can lead to heart dysfunction and an increased risk of heart failure if not properly managed. Humoral stimuli, such as inflammatory agents, oxidative stress, and mechanical stress triggers, in contrast, interact with cell surface receptors, initiating downstream second messenger pathways that ultimately lead to a hypertrophic cellular response and activation of related gene expression, leading to cardiac hypertrophy (46). Khan *et al*., in 2024, demonstrated the protective role of raspberry ketone against isoproterenol-induced cardiac hypertrophy in Wistar Rats. They observed that when rats were treated with raspberry ketone at 100 and 200 mg/kg, they reduced blood pressure and increased heart rate. Immunohistochemical studies reveal that it decreases the expression of caspase-3, nuclear factor-κB, tumor necrosis factor-α, and increases Peroxisome proliferator-activated receptor alpha (PPAR-α) receptor. Biochemical results indicate reduced cardiac markers: creatine kinase-MB and lactate dehydrogenase levels have decreased. Additionally, total cholesterol, triglycerides, and low-density lipoprotein levels have also decreased, while very-low-density lipoprotein levels have increased, along with high-density lipoprotein levels. In addition, it also affects the anti-oxidant markers, decreasing malondialdehyde and increasing glutathione, superoxide dismutase, catalase, Na+, and K+ -ATPase. Moreover, it also decreases the nitric oxide and the nitric oxide synthase level. This study confirmed the protective role of raspberry ketone against cardiac hypertrophy (Figure 1) (47). Another study explored the effects of blackberry (BL), raspberry (RB), and black raspberry (BRB) polyphenol extracts on mitigating Ang II-induced aging in vascular smooth muscle cells (VSMCs) and aimed to identify the underlying molecular pathways. Treatment with BL, RB, and BRB polyphenol extracts (200 µg/ml) reduced Ang II-induced senescence, evidenced by fewer cells positive for senescence-associated β-galactosidase (SA-β-gal) and reduced expression of p21 and p53, which correlated with lower reactive oxygen species (ROS) levels and inhibited Ang II signaling. The BL polyphenol extract boosted superoxide dismutase (SOD) 1 expression, reduced Nox1 expression, and inhibited the phosphorylation of Akt, p38MAPK, and ERK1/2 triggered by Ang II, while also decreasing senescence linked to Nox1 overexpression. On the other hand, RB and BRB extracts enhanced SOD1, SOD2, and glutathione peroxidase 1 (GPx1) expression, but did not affect Nox1 expression or senescence caused by Nox1 overexpression (Table 1). This study provides evidence that BL polyphenols mitigate Ang II-induced senescence via a Nox1-dependent pathway, while RB and BRB polyphenols reduce senescence through a Nox1-independent mechanism, likely by enhancing the cell’s anti-oxidant capacity (48).

### Raspberry’s potential role in neuroinflammatory diseases

**Figure 2:**
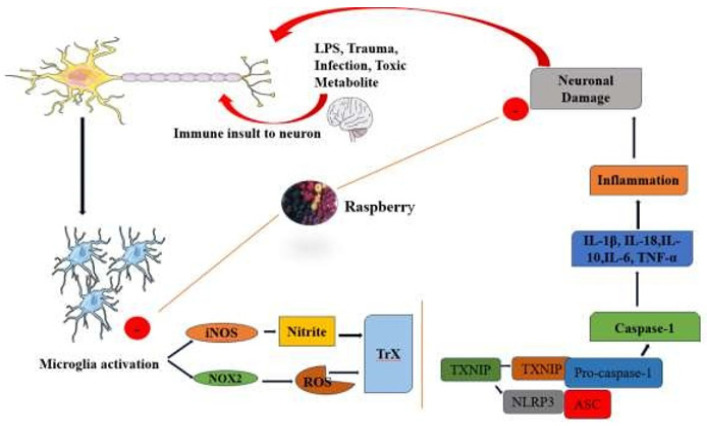
Mechanistic insight of raspberry in neuroinflammatory diseases

The global prevalence of neurological disorders like Alzheimer’s (AD) and Parkinson’s (PD) disease is increasing day by day, despite lifestyle modification and therapeutic approaches (49). Microglia, the immune cells of the central nervous system (CNS), typically remain in a homeostatic “resting” state, continuously monitoring for threats (50). However, upon detecting pathogens, microglia become activated, leading to significant changes in key inflammatory pathways. This results in up-reguation of cell-surface receptors and cytokines, such as tumor necrosis factor-alpha (TNF-α) (51). This proinflammatory response is closely associated with the neuroinflammation observed in neurodegenerative conditions like AD and PD (Figure 2) (52).

A study reported that treating N9 murine microglial cells with lipopolysaccharide (LPS) triggers microglial activation and activates inflammatory pathways. However, treatment with raspberries rich in ellagitannins and ellagic acid derivatives was found to suppress the activation of key inflammatory pathways, including CD40, NO, TNF-α, MAPK, NFAT, and NF-kB. This study suggests that these raspberry components may offer potential benefits for neuroinflammatory-related dysfunctions (53). Another study reported that murine BV-2 microglial cells were activated by LPS treatment. Researchers evaluated the potential effects of raspberry extract, including its polyphenols, and the gut-derived metabolite urolithin A (UroA) as treatments on LPS-induced BV-2 microglial cells. They discovered that both extracts reduced proinflammatory cytokine gene expression and inhibited the c-Jun N-terminal kinases (JNK) pathway, along with the subsequent p-c-Jun protein (JNK/c-Jun) signaling (54). This study also provides evidence that raspberries have a neuroprotective potential. Mu Tang and colleagues investigated the protective effects of black raspberry (BRB) anthocyanins against LPS-induced neuroinflammation in BV2 microglial cells. Their findings revealed that BRB treatment reduced the expression of NOX2 and its downstream molecules, including thioredoxin-interacting protein (TXNIP) and the NOD-like receptor protein 3 (NLRP3) inflammasome. Additionally, BRB anthocyanins suppressed the release of Interleukin-18 (IL-18) and Interleukin-1β (IL-1β), leading to a reduction in the inflammatory response triggered by LPS in BV2 microglia (Table 2). These findings highlight the potential of BRB anthocyanins as a promising preventive or therapeutic approach for neurodegenerative diseases in the future (55) (Figure 2). The gut microbiota contributes to maintaining gastrointestinal (GI) tract homeostasis and is thought to influence brain development and function in various ways. The two-way communication between the gut microbiota and the brain is known as the microbiota–gut–brain axis. This connection between the gut and brain appears to affect human health and behavior, with animal studies showing links between changes in the gut microbiota and neurological disorders (56). Research also highlights the current roles of raspberry polyphenols in supporting neurological stability by influencing the composition and metabolism of the gut microbiota and modulating signal transmission along the gut-brain axis, thereby reducing oxidative stress caused by ROS imbalance in the body, which is crucial for treating these neurodegenerative diseases (Figure 2) (57).

### Raspberry role in cancer

**Figure 3:**
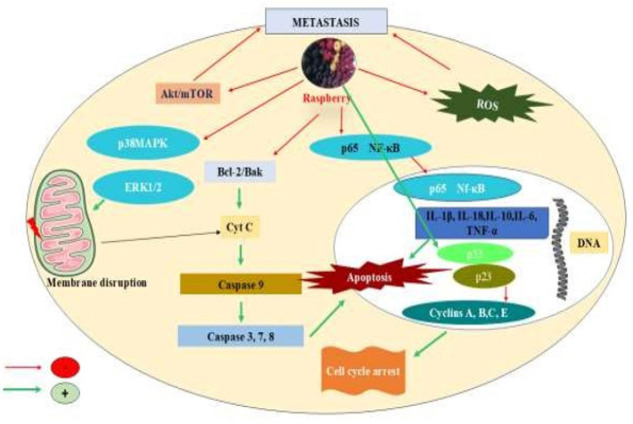
Potential effects of raspberries on cancer

Cancer is a disease characterized by the uncontrolled growth of certain cells in the body, which can spread to other areas. As cancer develops, cancer cells can leverage oxidative stress to further their growth (58). One of the most significant variables that contributes to the development of cancer is oxidative stress. Reactive oxygen species (ROS) can modify several signal transduction pathways, such as NF-κB, p53, MAPKs, P13K, HIF-1α, and NRF2, which can affect cell proliferation, cause DNA damage, and govern tumor angiogenesis (Figure 3). Certain cancer cells have developed the ability to thrive in high reactive oxygen species (ROS) environments by enhancing their anti-oxidant defence mechanisms. This enables them to flourish in environments that would typically be detrimental to normal cells. An oxidative environment can occasionally increase resistance to radiation and chemotherapy, which use ROS to destroy cancer cells (59). Anthocyanins and ellagitannins are the main anti-oxidant-producing components of raspberries, making up 25% and 52% of their total anti-oxidant capacity, respectively. These substances demonstrate their potential for cancer prevention by supporting the fight against oxidative stress, a significant contributor to the onset and spread of cancer (Figure 3) (60).

Studies conducted both *in vitro *and *in vivo* have shown that raspberries have anticancer properties. Numerous anticancer actions have been demonstrated, such as preventing malignant cell cycle progression, triggering apoptosis, preventing migration and angiogenesis, preventing proliferation, and repairing DNA damage (61).

The up-reguation of anti-apoptotic markers, such as Bcl-2 and Bcl-xl, along with the down-reguation or inactivation of pro-apoptotic markers, including caspase-3,8,9 and p53 enables cancer cells to evade programmed cell death, thereby facilitating uncontrolled growth and survival (Figure 3) (62).

Resveratrol, an active constituent of the raspberry, has anticancer activity against many cancers. It has shown significant cytotoxicity, decreasing the viability of MCF-7 breast cancer cells by 57.5% and HepG2 liver cancer cells by 56.2%. Additionally, it decreased anti-apoptotic mRNA levels of Bcl-2 and Bcl-xL while increasing the expression of apoptotic markers (caspase-3, -8, -9, Bax, p53, and p21), suggesting that it may have anticancer properties (63). In another study, raspberries reduced cell viability and triggered apoptosis in MCF-7 breast cancer cells by increasing reactive oxygen species (ROS) levels, resulting in mitochondrial damage and the up-reguation of apoptotic proteins, including caspase-9, p53, and Bax (Table 3) (64). Similarly, Madhusoodhanan and coworkers studied the effects of black raspberry extract (BRE) on the MCF-7 breast cancer cell line. They found that BRE effectively inhibited cancer cell proliferation by blocking NFκB activity. This inhibition of NFκB enhanced the apoptotic response and reduced the growth and clonogenic survival of various human cancer cell lines (Figure 3) (65).

In addition to their *in vitro *effects in breast cancer, studies involving *in vivo* models have demonstrated the cancer-fighting potential of raspberries in esophageal cancer (Table 3). In a 30-week experiment, the inclusion of 5% red raspberry in the diet significantly reduced the incidence of esophageal cancer induced by N-nitrosomethylbenzylamine in male F344 rats (66). In addition to their effects on breast cancer and esophageal cancer, raspberries also exhibit promising activity against head and neck squamous cell carcinoma. Black raspberry extract has been shown to reduce tumor growth in mouse models by enhancing immune responses, inhibiting regulatory T cell recruitment, and boosting CD8+ T cell activity at tumor sites. Additionally, it lowers PD-L1 expression in lymph nodes, suggesting its potential for cancer prevention and treatment (67). Raspberry extract’s anticancer action on HeLa cells was linked to increased production of the pro-apoptotic molecule tumor necrosis factor receptor superfamily member 6 (FAS) and the overexpression of the antiproliferative molecule P53 (68).

Black raspberry also has anticancer activity against oral cancer. The effect of black raspberry extract (BRE) on 4-nitroquinoline 1-oxide carcinogen-induced oral cancer experimental model has been studied to explore the role of the oral glucocorticoid (GC) system in oral cancer. The findings demonstrated that oral cancer has down-reguated HSD11B2, an enzyme that deactivates cortisol, which accelerates the growth of the tumor (Table 3). In comparison to synthetic glucocorticoids, BRE treatment raised HSD11B2 expression, decreased active GCS, and indicated a possible role for natural products in oral cancer chemoprevention (69). In another study, BREs slowed tumor progression in a rat model and reduced the formation of oral lesions by 28.6–39.3%. The chemopreventive impact was associated with decreased cellular proliferation, anti-apoptotic activity, and inflammation (70). Mallery and associates assessed a bioadhesive gel with 10% freeze-dried black raspberry (BRB) over three months, finding significant clinical regression of lesions, reduced histological grades, and decreased loss of heterozygosity (LOH) events, all without side effects (71).

In addition to the previously discussed anticancer properties of raspberries, a study on jaboticaba peel extract, a Brazilian berry, demonstrated anticancer effects in LNCaP and PC-3 prostate cancer cells. This was achieved by modulating NF-κB and promoting apoptosis through the regulation of B-cell lymphoma 2 (Bcl-2) and BAX (Figure 3) (72).

Beyond the previously noted anticancer properties of raspberries, studies investigating the chemical components of black raspberries, blueberries, and blackberries have explored their effects on human A549 lung cancer cells. Volatile extracts from these berries demonstrated potent anticancer activity by down-reguating inactive apoptotic proteins such as poly (ADP-ribose) polymerase (PARP), procaspase-9, and procaspase-3 (Table 3). This suppression triggered increased apoptotic cell death and induced G0/G1 phase cell cycle arrest in lung cancer cells (73). Further expanding on the anticancer properties of raspberries, red raspberry extract has been shown to inhibit the proliferation of liver cancer cells while promoting apoptosis. This effect is achieved by decreasing the Bcl-2/Bax protein ratio and lowering mitochondrial membrane potential (74).

However, no evidence has been reported indicating side effects of raspberries, though further research is needed to confirm their safety as a supplement (75). In a 2016 clinical trial, An *et al*. observed that participants in the high-dose raspberry–treated prediabetic group experienced side effects such as insomnia, nausea, and skin rashes (76). Some studies have examined the potential toxicity of raspberry leaves, which pregnant women traditionally use to ease childbirth. Findings show no significant adverse effects, with toxicity observed only in animal models where very high doses of raspberry leaf extract were administered intravenously (77-79). Researchers also reported cyto- and genotoxic effects of a red raspberry ellagitannin preparation (REP) within the concentration range of 2.5–160 μg/ml, along with its major individual ellagitannins, sanguiin H-6 (SH-6, 12.8–256 μM) and lambertianin C (LC, 9.3–378 μM), in human colon adenocarcinoma Caco-2 cells. The tested concentrations reflect levels naturally present in raspberry-containing foods. REP, SH-6, and LC demonstrated pronounced concentration-dependent genotoxicity, resulting in DNA damage between 7.3 ± 1.3% and 56.8 ± 4.3%, including double-strand breaks and oxidative modifications of DNA bases. At its IC_50_ value (124 μg/ml), REP altered nuclear morphology and triggered apoptosis in Caco-2 cells, and observed chemopreventive potential (80).

Expanding on the previously discussed anticancer properties, ellagitannins found in red raspberries have been shown to promote apoptosis in tumor cells effectively. Ellagitannin in red raspberry also promoted tumor cell apoptosis. Apoptosis of Caco-2 cells was induced by red raspberry ellagitannin preparation (40–160 µg/ml), sanguiin H-6 (26.7–256 µM), and lambertianin C (18.9–378 µM) (78). After a 24-hr treatment with 40 µM of sanguiin H-6, A2780 human ovarian carcinoma cells exhibited activation of the p38 signaling pathway, which promotes apoptosis. This indicates that sanguiin H-6 may possess therapeutic potential for inducing programmed cell death in ovarian carcinoma cells (81).

### Preclinical and clinical studies

Ding *et al.* demonstrated through both *in vitro* studies using HUVECs and *in vivo* experiments in C57BL/6 mice that the beneficial actions of ellagic acid are likely associated with Nrf2 activation and enhanced eNOS activity, resulting in vasodilation and reduced oxidative stress. Collectively, these effects contribute to improved endothelial function, which may help lower the risk of hypertension and atherosclerosis (82). In another study, normal and spontaneously hypertensive rats received red raspberry extract at doses of 100 or 200 mg/kg/day for 5 weeks. The treatment led to a dose-dependent decrease in blood pressure in hypertensive rats, accompanied by increased NO activation, reduced endothelin-1 levels, dose-specific anti-oxidant activity, and enhanced vascular endothelial function Table 1 (83).

In a clinical study, Myung *et al*. found that supplementing with black raspberry over 12 weeks significantly lowered serum total cholesterol, inflammatory cytokines, and augmentation index, thereby enhancing vascular endothelial function in statin-naïve individuals with metabolic syndrome (84). Similarly, another study demonstrated that consuming 38 g of raspberry powder daily (equivalent to 250 g of rehydrated berries) for six weeks in sedentary adult males and females boosted natural killer (NK) cell activity and plasma redox capacity, while also reducing blood pressure, augmentation index (AIx), central pulse wave velocity, and aortic systolic pressure (85). In a clinical trial investigating the effects of a raspberry intervention that included black raspberries, researchers observed a significant reduction in blood pressure among prehypertensive individuals. Over eight weeks, participants who regularly consumed black raspberries showed notable improvements in their 24-hr ambulatory blood pressure measurements. This suggests that raspberries may play a beneficial role in cardiovascular health by contributing to sustained blood pressure control (86). The findings highlight the potential of incorporating raspberries into the diet as a natural, dietary approach to managing prehypertension and reducing the risk of progression to hypertension.

Researchers reported that spinal cord injury in rats led to Alzheimer’s-like symptoms within 14 days. Treatment with raspberry anthocyanin extract at a dose of 400 mg/kg reduced lesion volume, minimized neuronal loss, alleviated oxidative stress, and improved motor neuron function Table 2 (87).

Black raspberry (BRB) anthocyanins, combined with either 5-fluorouracil (5-FU) or Celecoxib, showed strong synergistic effects in preventing tumor formation and cancer cell proliferation in a preclinical model of colorectal cancer. MDR1 expression reversal, AKT signalling suppression, PTEN overexpression, and EZH2 down-reguation were mechanistically linked to this increased anticancer activity (88). Interestingly, while BRB components show therapeutic promise in colorectal cancer, their effects in other cancer types warrant careful consideration. For instance, Ellagic acid, a compound in BRB, may interfere with chemotherapy for castrate-resistant prostate cancer by affecting tubulin function and drug efflux in an animal model. However, BRB as a whole does not impact chemotherapy effectiveness and appears safe to consume during treatment (89).

It is reported that many clinical studies have been conducted in cancer patients to explore the effect of raspberry. For instance, in a study on men with localized prostate cancer, black BRB confections and nectar were found to be safe, well-tolerated, and highly adherent. Both forms delivered bioavailable phytochemicals, with the confection showing greater metabolite excretion (90). *In vitro *findings indicate that raspberry oil treatment in LoVo, MCF-7, and A549 cell lines suppressed cancer cell proliferation, reduced cell viability, and induced DNA damage, effects associated with elevated nitric oxide levels (91).

In another clinical investigation, a phase I study was conducted in patients with colorectal cancer (CRC) to assess the molecular effects of BRB supplementation. Participants received 60 g of freeze-dried BRB powder daily for nine weeks. The study demonstrated significant modulation of the Wnt signaling pathway, including demethylation of tumor suppressor genes SFRP2 and WIF1, as well as reduced expression of β-catenin and E-cadherin (92- 94).

### Implications and Limitations

Although preclinical studies strongly suggest that raspberries and their bioactive compounds possess anti-inflammatory potential, several challenges must be resolved before these findings can be effectively translated into clinical practice. Most existing research has been carried out *in vitro *on neuronal, cardiac, and cancer cell lines or in animal models, mainly mice. While these models provide important mechanistic insights, they do not fully capture the complexity of human cardiovascular, neuronal, and cancer systems, limiting their direct clinical relevance. Moreover, the pharmacokinetics of raspberry-derived compounds in humans remain poorly understood, with significant variability between individuals.

The chemical properties of raspberries can vary depending on the cultivar, environmental factors, and processing methods, which may influence the uniformity and potency of their anti-inflammatory effects.

At present, there is a shortage of large, rigorously designed randomized clinical trials assessing the anti-inflammatory potential of raspberry bioactives. The available human data are mostly limited to small pilot trials or observational studies, which typically involve whole raspberries or extracts and focus on biomarkers such as oxidative stress rather than direct cardiovascular, neuroinflammatory, and cancer outcomes. These findings, while indicative, are insufficient to establish causality or develop clinical guidelines. Moreover, the appropriate dosage, formulation, and mode of delivery of raspberries’ active constituents for therapeutic use have yet to be determined. Their possible interactions with conventional cardiovascular, neuroinflammatory, and cancer treatments, as well as their long-term safety for patients, also remain inadequately explored.

To translate laboratory findings into clinical applications, several key strategies are needed. Standardization of raspberry extracts with consistent potency and well-characterized levels of major bioactive compounds is a priority. Human pharmacokinetic and bioavailability studies are essential for clarifying absorption, metabolism, and excretion patterns and informing effective dosing regimens. Large-scale, rigorously designed randomized controlled trials across diverse populations and inflammatory conditions, including cardiovascular, neuroinflammatory, and cancer-related diseases, are critical to confirm efficacy and safety. Moreover, the potential synergistic effects of raspberry bioactives with conventional treatments such as lipid-lowering and antihypertensive agents, neuroprotective drugs, chemotherapy, radiotherapy, and immunotherapy warrant investigation. Preclinical studies should continue to elucidate the underlying mechanisms of these interactions to support their future incorporation into anti-inflammatory treatment strategies.

Activation of different signaling pathways, such as stimulation of oxidative stress, releases various ROS. Activation of Ag-II further stimulates NOX1, ROS, P-AKT/P-P38/ERK1/2, PPAR-α oxidative, NF-κB, which results in the release of multiple inflammatory cytokines, adhesion molecules, and caspase activation, leading to impaired blood flow, cardiac dysfunction, atherosclerosis, and myocardial infarction. Raspberry inhibits these pathways and improves cardiac dysfunctions.

Ag-II: Angiotensin-II, NOX1: NADPH oxidase 1, Nicotinamide adenine dinucleotide phosphate oxidase, ROS: Reactive Oxygen Species, PPAR-α: Peroxisome proliferator- activated receptor alpha, P-AKT/P-P38/ERK1/2: Akt signaling pathway/Extracellular signal- regulated kinases, CVS: Cardiovascular system, NF-κB: Nuclear factor kappa- B cells, TNF- α: Tumor necrosis factor alpha, IL-6: Interleukin 6, IL-1β: Interleukin-1 beta.

**Table 1:** Effects of raspberries on cardiovascular diseases

Type of cancer	Bioactive compound /raspberry used	*In* *vitro/in vivo* studies	Outcome	Molecular target	Ref.
Myocardial infarction	Raspberry	Wistar rats	↓ CKMB, TC, LDL, VLDL, ↑ HDL	↓NO, TNF-α, iNOS	43
Atherosclerosis	Ellagic acid	Umbilical vein endothelial cells	↓ IL-1β, E-selectin, VCAM	↓ NF-kB, CAMS, ROS	44
Cardiac hypertrophy	Raspberry	Wistar rats	↓ CKMB, MDA TC, LDL, VLDL, ↑ HDL, SOD	↓Caspase-3, NF- kB, TNF-α and ↑PPAR-α	47
Vascular smooth muscle cells affect	Raspberry and black raspberry polyphenol	Vascular smooth muscle cells	↓Nox1, ROS, ↑ SOD, GPx1	Inhibit Ang-II, Phosphorylation of Akt, p38MAPK, and ERK1/2	48
Atherosclerosis and hypertension	Raspberry	HUVEC/C57BL/6 mice	↓Oxidative stress, ↑ Vasodilation	↑Nrf2, eNOS	80
Hypertension	Raspberry	Wistar rats	↓Oxidative stress, ↑Vasodialation	↑NO, Improve endothelium functions	81

Ag-II: Angiotensin-II, NOX1: NADPH oxidase 1, Nicotinamide adenine dinucleotide phosphate oxidase, ROS: Reactive Oxygen Species, PPAR-α: Peroxisome proliferator- activated receptor alpha, P-AKT/P-P38/ERK1/2: Akt signaling pathway/Extracellular signal- regulated kinases, NF-κB: Nuclear factor kappa- B cells, TNF-α: Tumor necrosis factor alpha, IL-6: Interleukin 6, IL-1β: Interleukin-1 beta, eNOS- Endothelial nitric oxide synthase, Nrf2: Nuclear factor erythroid 2-related factor, CAMs: Cell adhesion molecules, HUVECs: Human umbilical vein endothelial cells, CKMB: Creatine kinase-MB, NO: Nitric Oxide, iNOS: inducible nitric oxide synthase.

LPS, Trauma, infection, and toxic metabolites activate the neuron microglia. It activates the different neuronal signaling pathways, such as stimulation of iNOS and NOX, which trigger oxidative stress, and thioredoxin and caspase 1, which result in the release of multiple inflammatory cytokines, leading to neuronal inflammation and neuronal damage. Raspberry inhibits these pathways and reflects neuroprotective effects.

IL-1β: Interleukin-1 beta, Interleukin, NOX2: NADPH oxidase 2, cytochrome b(558) subunit beta or Cytochrome b-245 heavy chain, Nicotinamide adenine dinucleotide phosphate oxidase, iNOS: Inducible nitric oxide synthase, ROS: Reactive Oxygen Species, TrX: Thioredoxin, TXNIP: Thioredoxin Interacting Protein, NLRP3: NLR family pyrin domain containing 3; IL- 1β: Interleukin-1 beta, IL-1α: Interleukin-1 alpha, IL-10: Interleukin-10, IL-18: Interleukin-18; TNF-α: Tumor necrosis factor alpha.

**Table 2:** Effects of raspberries on neurodegenerative diseases

Type of cancer	Bioactive compound /part of raspberry used	*In* *vitro*/ *in vivo* studies	Outcome	Molecular target	Ref.
Neuroinflammation	Ellagitannins and ellagic acid	N9 murine microglial cells	↓CD40, NO, TNF-α, NFAT	↓ NF-kB, MAPK	53
Neuroinflammation	Raspberry extract polyphenols	Murine BV-2 microglia cells	↓TNF-α, IL-6	Inhibit the c-Jun N-terminal kinases	54
Neuroinflammation	Black raspberry (BRB) anthocyanins	BV2 microglial cells	↓TXNIP, NLRP3, IL- 18, IL-1β	↓NOX2	55
Alzheimer	Raspberry extract anthocyanins	Spinal cord injury rats	↓Lesion volume, neuronal loss	↑ Motor neuron cells, ↓oxidative stress	87

NO: Nitric oxide, ROS: Reactive Oxygen Species, TrX: Thioredoxin, TXNIP: Thioredoxin Interacting Protein, NLRP3: NLR family pyrin domain containing 3; IL-1β: Interleukin-1 beta, IL-1α: Interleukin-1 alpha, IL-10: Interleukin-10, IL-18: Interleukin-18; TNF-α: Tumor necrosis factor alpha., NFAT: Nuclear factor of activated T-cells, MAPK: mitogen-activated protein kinase, NOX1: NADPH oxidase 1

Raspberry inhibits cancer cell proliferation primarily by blocking the NF-κB signaling pathway, thereby reducing the release of inflammatory cytokines. It also suppresses the activation of p38 MAPK and ERK pathways. Additionally, raspberry induces apoptosis in cancer cells by down-reguating Bcl-2 expression and activating caspase-9. It further halts cancer cell cycle progression by activating the tumor suppressor gene p53 and impedes metastasis by inhibiting the Akt/mTOR signaling cascade. These anticancer effects are primarily linked to the raspberry's potent antioxidant properties, which help lower intracellular reactive oxygen species (ROS) levels.

NF-kB: Nuclear factor kappa B, p38MAPK: p38 mitogen activated protein kinase, ERKs: extracellular signal-regulated kinases, Bcl-2: B-cell lymphoma 2, IL-6: Interleukin, IL-1β: Interleukin-1 beta, IL-10: Interleukin 10, IL-18: Interleukin-18, ROS: Reactive Oxygen Species, TNF-α: Tumor necrosis factor alpha, Cyt C: cytochrome complex.

**Table 3:** Effects of raspberries on different types of cancer diseases

Type of cancer	Bioactive compound /part of raspberry used	*In* *vitro*/ *in vivo* studies	Outcome	Molecular target	Ref.
Head and neck squamous cell carcinoma	Black raspberry extract	C57Bl/6 mice	PD-L1 expression by Myeloid Cells, Enhancement of CD8+ T-cell activity↓	PD-L1 ↑	67
Cervical cancer	Raspberry extract	Hela cell	Inhibition of cell proliferation and induces apoptosis	P53, FAS ↑	68
Oral cancer	Black raspberry extract	CAL27 cell	Inhibition of cell proliferation, angiogenesis and invasion	EGFR, VEGFA ↓	69
Lung cancer	Volatile extract of black raspberries, blueberries, and blackberries	A549 lung cancer cells	Induction of apoptosis and cell cycle arrest at G0/G1 phase	PARP, procaspase-9, and procaspase-3↓	73
Liver cancer	Red raspberry extract	HepG2 cells	Inhibit cell proliferation and promote apoptosis	Bcl-2/Bcl-2-associated x (Bax) protein ratio ↓ ROS ↑	74
Ovarian cancer	Ellagitannin	A2780 cell	Apoptosis and cell death ↑	p-38 ↑	81
Breast cancer, colon adenocarcinoma and lung cancer	Raspberry oil	LoVo, MCF-7 and A549 cell lines	Inhibition of cancer cell line proliferation, cell viability, and DNA damage in cancer cell	ROS and NO↑	91
Colorectal cancer	Red raspberry	C57BL/6J mice	Cell proliferation ↓	E-cadherin↑ β-catenin and mucin 1↓	93

PD-L1: Programmed death-ligand 1, Bcl-2: B-cell lymphoma 2, IL-6: Interleukin, ROS: Reactive Oxygen Species, TNF-α: Tumor necrosis factor alpha, VEGF: Vascular endothelial growth factor A, EGFR: Epidermal Growth Factor Receptor, PARP: Poly (ADP-ribose) polymerase, BAX: Bcl-2-associated X protein.

## Conclusion

Extensive research has highlighted the therapeutic potential of raspberries, which are rich in ellagitannins, ellagic acid, anthocyanins, and polyphenolic compounds, in combating cardiovascular diseases, neuroinflammation, and cancer. Their notable anti-inflammatory, anti-oxidant, and cytoprotective effects help regulate vital signaling pathways, such as NF-κB and MAPK, reduce oxidative stress, and modulate immune responses. These actions underline their importance in both preventing and supporting the treatment of chronic conditions driven by inflammation and cellular injury. Further investigations are essential to identify the most effective bioactive components in raspberries and to optimize their clinical application with minimal side effects.

Future investigations should prioritize several key areas. Firstly, large-scale randomized clinical trials are needed to assess the efficacy and safety of the active constituents of raspberries in cardiovascular, neuroinflammatory, and cancer prevention and treatment. These trials should involve diverse patient populations to ensure broader applicability of the results. Secondly, detailed studies on the bioavailability and metabolic pathways of raspberry-derived compounds in humans are essential to understand their therapeutic potential better. In addition, it is important to investigate how raspberry bioactives may interact with standard cardiovascular, neuroinflammatory, and cancer treatments. Lastly, personalized strategies that account for genetic, metabolic, and microbiome variations could help maximize the therapeutic benefits of raspberry bioactives.

## Declaration

We have not used any AI tools or technologies to prepare this manuscript.
